# Rifle Shooting Performance Correlates with Electroencephalogram Beta Rhythm Network Activity during Aiming

**DOI:** 10.1155/2018/4097561

**Published:** 2018-11-11

**Authors:** Anmin Gong, Jianping Liu, Changhao Jiang, Yunfa Fu

**Affiliations:** ^1^School of Information Engineering, Engineering University of Armed Police Force, Xi'an, China; ^2^Key Laboratory of Sports Performance Evaluation and Technical Analysis, Capital Institute of Physical Education, Beijing, China; ^3^School of Information Engineering and Automation, Kunming University of Science and Technology, Kunming, China

## Abstract

To study the relationship between brain network and shooting performance during shooting aiming, we collected electroencephalogram (EEG) signals from 40 skilled shooters during rifle shooting and calculated the EEG functional coupling, functional brain network topology, and correlation coefficients between these EEG characteristics and shooting performance. Our result shows a significant negative correlation between shooting performance and functional coupling between the prefrontal, frontal, and temporal regions of the right brain in the Beta1 and Beta2 frequency bands. Global and local brain network topology characteristics were also significantly correlated with shooting performance. These findings indicate that under these experimental conditions, shooters with higher shooting performances exhibit lower functional coupling, higher global, and lower local information integration efficiency during shooting. These conclusions may provide a theoretical basis of the EEG brain network for studying the mental status of shooters while shooting.

## 1. Introduction

In the field of motor neuroscience, the neural efficiency hypothesis is a crucial research hotspot; this hypothesis was initially applied to the study of cognitive tasks, and then extended into the field of sports Science [1–4]. Hatfield and Kerick detailed how the neural efficiency is reflected in psychomotor behavioral and suggested that less complexity in the processes associated with motor control or a reduction in the degrees of freedom of relevant neural network actions may lead to greater consistency of the resultant motor performance [5]. Consequently, more skilled athletes tend to have lower levels of brain activity than novice during the motor behavior. In addition, as athletes become more skilled, these processes could mediate the reduction in the widespread activity in regions mapping executive control; and could result in a shift toward more automated processing [6]. This phenomenon could be attributed to the neuroplasticity caused by long-term professional skill training, that is, marked changes in the structure and function of the brain of the skilled athletes compared with the ordinary untrained subjects [7]. Thus, the neural efficiency hypothesis can be reflected in neural signals (such as electroencephalogram (EEG), forming some specific neural markers that are closely associated with the motor performance; the most common EEG markers include band power characteristics and vent-related desynchronization/event-related synchronization (ERD/ERS) characteristics [8, 9].

By studying these neural markers closely related to motor performance, we can evaluate the level of athletes and guide the training, resulting to improve motor performance. For example, elite gymnasts and karate athletes exhibit lower amplitude of frontoparietal Alpha ERD during movement, compared to novices [10]. During eyes closed resting states, elite karate athletes exhibit higher amplitude parietal and occipital Alpha1 band power compared to novices [11]. In the field of shooting, Hatfield et al. found that when the firing time approached, the Alpha band power in the left temporal regions of experts continued to increase [12, 13]. Del Percio et al. found that the ERD amplitude in the Alpha and Beta bands, during the aiming phase, was significantly lower than that observed in novices [14]. Hung et al. found that skilled shooters had increased power in the Alpha1, Alpha2, and Beta1 bands during actual aiming compared to a postural control condition [15]. Furthermore, di Fronso et al. divided the shooting movement into four types based on the shooting performance and level of action control and investigated the EEG ERD/ERS activity during different types of shooting aiming. This method provides more accurate support and more targeted neural markers for the study of motor performance [16]. In addition to the traditionally analysis, some scholars studied EEG changes during shooting from the perspective of brain functional coupling. Deeny et al. analyzed differences in EEG coherence and phase shift characteristics between experts and novices and found that Alpha and Beta band coherence between the right frontal, and other brain regions, was lower in expert shooters, compared to novices [17, 18]. Del Percio et al. proposed the concept of Event Related Coherence (ERCoh), and they found that the experts' intrahemispheric Alpha1 (parietal-temporal and parietal-occipital regions), Beta2, and Gamma (parietal-temporal regions) ERCoh exhibited more stable amplitude than novices during the preparation of pistol shots [19].

In recent years, neuroscientists have suggested that the brain is an efficient, sparse, small-world network of regions that perform different functions. When performing different tasks, discrete brain areas coordinate with each other to complete a series of simple or complex jobs [20–22]. Investigatory methods based on brain networks have several advantages in terms of their practical application: they can both analyze the cooperative work of different functional areas of the brain and reveal dynamic changes in topology characteristics in participants performing tasks. Therefore, research based on this concept has attracted much attention [23–27].

In summary, although EEG dynamics during shooting aiming has been previously investigated, a comprehensive study using brain network methods to analyze EEG data is lacking. To date, the dynamics of the brain network during the aiming period and whether the functional brain network during the aiming period is related to shooting performance are unknown. At the same time, we also do not know which specific frequency bands, brain regions, connection, and network topology characteristics are most closely related to shooting performance. Therefore, we hypothesize that the brain network, especially functional coupling characteristics and brain network topological characteristics, is closely linked to shooting performance in skilled shooters. To test this hypothesis, we collected EEG signals from 40 shooters over the entire shooting process and performed a correlation analysis between EEG functional coupling, characteristics of brain network topology, and shooting performance.

## 2. Materials and Methods

### 2.1. Subjects

The experiment was conducted at an armed police training base in China Xi'an; 40 healthy subjects (Chinese armed police officers, men, age: 20 ± 2 years) took part in this experiment. Individuals were eligible to participate if they

- had no neurologic disorders, such as epilepsy,
- had never experienced a major head injury resulting in coma or craniotomy,
- pulled the trigger with the right hand and aimed with the right eye.

All subjects were trained for 2 years and attended at least two shooting sessions each week for 2 hours. According to the study time and training intensity, the shooting level can be judged as skilled shooters [28]. This experiment underwent review by the university ethics committee. Participants voluntarily took part in this research, understood the experimental process and purpose, and provided written informed consent to participate.

### 2.2. Experimental Procedures

**Figure 1:**
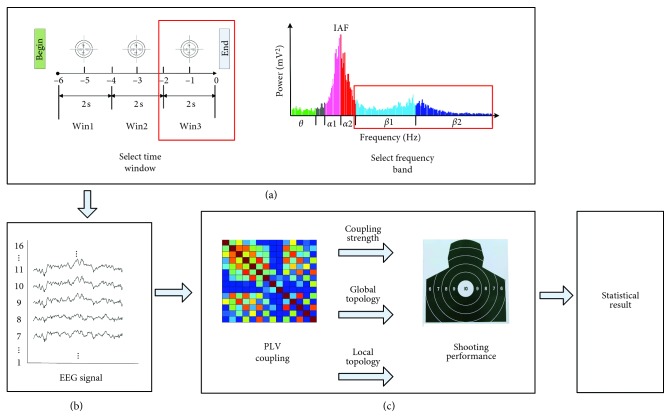
(a) The parameter selecting: frequency bands and time windows which most closely related to shooting performance were determined. (b) Signal processing: PLV values between each two EEG channels were calculated in the selected frequency band (Beta1 and Beta2) and the time window (Win3). (c) Correlation analysis: the correlation between PLV network characteristics and shooting performance was calculated. The above is the correlation analysis between the coupling strength and shooting performance. The median is the correlation analysis between the brain network global topology characteristics and shooting performance. The down is correlation analysis between brain network local topology characteristics and shooting performance.

EEG acquisition was divided into two parts: the resting state and the shooting stage. During the resting state, the subjects sat in a comfy seat, kept their eyes closed and eyes opened for an acquisition time of 5 min. During the shooting state, the subjects used a type 95 rifle with 5.8-mm caliber bullets, aiming at the outside of a target 100 m away. The target size is 52 × 52 cm with 10 ring diameter of 10 cm. Each extension of 5 cm from the edge of the 10 ring is 9, 8, 7, 6 ring (as shown in Figure 1). Every shooter takes a prone position, single firing mode, after firing the feedback score, the shooter adjusts the aiming point by themselves according to the score, and then performs the next shot. All subjects performed 60 shots at their own pace. Each shot time is recorded by a sound sensor and transmitted to the EEG acquisition device as the firing time tag (>90 dB). According to the rules of uniform shooting performance assessment of the Chinese armed police officers, target hits were scored from 6 to 10, while a miss being scored as 0.

### 2.3. EEG Acquisition and Preprocessing

**Figure 2:**
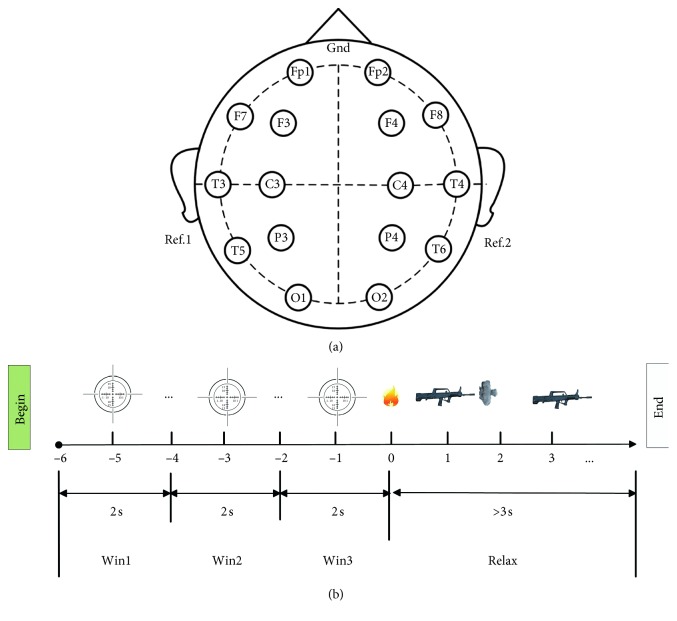
(a) The placement of electrodes using the standard 10–20 system (16 channels: Fp1, Fp2, F3, F4, C3, C4, P3, P4, O1, O2, F7, F8, T3, T4, T5, and T6. Forehead: ground, left, and right mastoid processes: references). (b) The experimental process.

In this study, we used a portable Holter-16D EEG amplifier (Symtop Instrument, Beijing, China). Electrode placement was in accordance with the international 10–20 system, comprising 16 electrodes. Detailed electrode placement is illustrated in Figure 2(a). Data were recorded sampled at 1000 Hz and filtered online with a notch filter of 50 Hz.

After acquisition, EEG signals were first subjected to a band-pass filter of 0.1–50 Hz band-pass filtering by EEGLAB toolbox [29]. According to the position of the firing time, the EEG data of 6 s before firing and 2 s after firing was selected. It was recorded as a trial. Remove the trails that affected by EMG or body movements influence (remove rate ≈ 17%). Finally, 38 subjects (two subjects were removed) and 1904 usable trials were obtained (each subjects have about 50 trials).

Alpha power in the closed eye resting-state EEG was computed by the occipital area to define each subject's individual alpha frequency (IAF). After being tested in accordance with the definition of each IAF frequency range: theta frequency ranges as IAF − 6 Hz to IAF − 4 Hz, alpha1 as IAF − 2 Hz to IAF, alpha2 as IAF to IAF + 2 Hz, Beta1 2 as IAF + 2 Hz to 20 Hz, and Beta2 as 20 to 30 Hz [29]. Due to shoot is a fine movement, neural activity before aiming changes rapidly. Therefore, we define the firing time tag as 0 s and divide the EEG before firing time tag into three time windows. Each two second time window was defined as Win1 (−6 s to −4 s), Win2 (−4 s to −2 s), and Win3 (−2 s to 0 s) (Figure 2(b)).

The analysis process is presented in Figure 1. First, the most suitable for the analysis frequency band and time window were determined. Then, EEG characteristics were extracted in the selected frequency band and the time window, including the coupling strength, global brain network topology, and local brain network topology. Final, correlation analysis between the EEG characteristics and shooting performance was performed, and the statistical results were obtained.

### 2.4. Functional Coupling Analysis

In this article, we use the phase locking value (PLV) method to calculate the functional coupling of each EEG signals pairs [31, 32]. The calculation process comprises the following steps [33]. First, depending on the IAF and band division of each subject, Butterworth band-pass filters with different frequency bands are constructed respectively. Then, EEG signals of each subject are band-pass filtered, and the filtered EEG signals are obtained at a given frequency band. Second, perform the Hilbert transformation to the filtered EEG. Then, the phase value of the signal is computed according to the original signal and the transformed conjugate signal. Finally, according to the phase values of the two sets of signals, the average value of the phase difference between two signals is calculated [34]. The above calculation process was also implemented within the MATLAB 2014 platform.

The EEG data format after preprocessing was 38 × 50 × 16 × 6000, representing 38 subjects, 50 trails, 16 channels, and 6000 data samplings. In this paper, we mainly study the EEG signals during Win3 window in the Beta1 and Beta2 bands (the detail seen result section). Finally, the connection matrices of subjects are obtained, and the data format is 16 × 16 × 2 × 38, representing the 38 subjects, 2 frequency bands, and the 16 × 16 is the connectivity matrices.

### 2.5. Topology Characteristics of Brain Network

For the functional coupling characteristics calculated, we respectively analyzed the following topology characteristics.

Coupling strength, the original connection value obtained from the PLV method, was averaged by the coupling values of all available trials for each subject. Then, based on the functional coupling matrix of each subject, without threshold, coupling value between all nodes was reserved, and the brain function weight network was obtained. We additionally performed the brain network topology analysis under this brain network condition.

Central to brain network analysis is graph theory analysis, which considers different regions of the brain as nodes and considers the connection relations as edges, and then uses graph theory to compute the global or local topological characteristics of the network [35].

In the global characteristics analysis, four characteristics are chosen: the clustering coefficient (*C*), mean local efficiency (*E*_l_), characteristic path length (*L*_c_), and global efficiency (*E*_g_).

The clustering coefficient is

(1) $$\begin{array}{c} C=\frac{1}{n}\underset{i\in N}{\sum }C_{i}=\frac{1}{n}\underset{i\in N}{\sum }\frac{2t_{i}}{k_{i}\left(k_{i}-1\right)}, \end{array}$$

where *C* is the clustering coefficient of all nodes and the clustering coefficient of each node (*C*_*i*_) represents the probability of a connection to a given neighboring node.

The mean local efficiency is

(2) $$\begin{array}{c} E_{l}=\frac{1}{n}\underset{i\in N}{\sum }E_{l,i}=\frac{1}{n}\underset{i\in N}{\sum }\frac{{\sum_{j,h\in N,j\neq i}a_{ij}a_{ih}\left[d_{jh}\left(N_{i}\right)\right]}^{-1}}{k_{i}\left(k_{i}-1\right)}, \end{array}$$

where *E*_l,*i*_ represents the local efficiency of node *i* and *d*_*jh*_(*N*_*i*_) represents the length of the shortest path between *j* and *h* that contains only neighbors of *i*. The mean local efficiency represents the average efficiency of all subnetworks (subnets) in the brain network and each subnet consists of all nodes directly connected to a given node [36].

The characteristic path length is

(3) $$\begin{array}{c} L_{c}=\frac{1}{n}\underset{i\in N}{\sum }L_{i}=\frac{1}{n}\underset{i\in N}{\sum }\frac{\sum_{j\in N,j\neq i}d_{ij}}{n-1}, \end{array}$$

where *L*_c_ is the average of the shortest path for all pairs of nodes. In the weight matrix, the length of the edge connection is the reciprocal of the connectivity value and the characteristic path of each pair of nodes (*L*_*i*_) is the shortest of all paths from node *i* to node *j* [37]. The characteristic path length measures the overall routing efficiency of the network.

The global efficiency,

(4) $$\begin{array}{c} E_{g}=\frac{1}{n}\sum_{i\in N}\frac{\sum_{j\in N,j\neq i}{\left(d_{ij}\right)}^{-1}}{n-1}, \end{array}$$

is the average of the inverse of the shortest path for all pairs of nodes. If the shortest path between nodes is smaller, global efficiency is higher. Alternatively, lower global efficiency indicates a longer, shortest path between nodes [36].

For local characteristics, choose the average coupling strength, local efficiency, eigenvector centrality, and the local clustering coefficient as four analysis characteristics.

The average coupling strength is the accumulate sum of the element of the matrix row or column. The calculation formula is

(5) $$\begin{array}{c} S_{i}=\overset{N}{\underset{j=1}{\sum }}CV_{i}\left(i,j\right), \end{array}$$

where *i* and *j* represent the node *i* and node *j*, and *N* represents the total number of nodes.

The local clustering coefficient reflects the probability of the connection between neighboring nodes connected to this node. The computational formula is shown as *C*_*i*_ in Formula (1).

The eigenvector centrality can reflect the importance of object degree synthetically. The formula is shown in

(6) $$\begin{array}{c} D_{i}=u\overset{n}{\underset{j=1}{\sum }}a_{ij}x_{j},Ax=\lambda x\text{ or equivalently},x=\frac{1}{\lambda }Ax, \end{array}$$

where *D*_*i*_ represents the eigenvector centrality of node *i*, *A* represents the adjacency matrix, *λ* represents the max eigenvalue, *x* represents the eigenvector, and *u* represents 1/*λ*. The eigenvector centrality of node *i* can be represented as the weighted sum of the eigenvector of the other nodes.

The local efficiency of the brain network reflects the transmission efficiency of the node graph. The computational formula is shown as *E*_*i*_ in Formula (2).

### 2.6. Statistical Analysis

We hypothesized that the functional coupling and network topology characteristics during Win3 window in Beta1 band and Beta2 band are correlated with the shooting performance of the subjects. To verify this hypothesis, we use the statistical analysis to analyze the correlation between characteristics and shooting performance. Each subject's shooting performance is evaluated by the average of 55 times shot's score. The average shooting performance of subjects was 8.8 ± 0.41. EEG characteristics and shooting performance were assessed for Gaussian distribution using the Kolmogorov–Smirnov test. The results show that shooting performance followed the Gauss distribution (*P* > 0.05), but not all EEG characteristics subjected the Gauss distribution. So, we choose the Spearman rank correlation analysis to perform the correlation analysis and calculate the correlation coefficients (*r*).

In order to decrease the influence of outliers, the outlier removal strategy was as follows. First, the linear regression equation of EEG characteristics and shooting performance was calculated for all the samples, according to the above rules. Then the distances between each sample point and the regression equation were calculated, and we removed the sample points of the 10% maximum distance (three points) from the regression equation. Finally, we calculated the correlation coefficients of the sample after removing the outliers, and the final correlation coefficients were obtained.

## 3. Results

### 3.1. Frequency Band and Time Window for Analysis

To determine the frequency band and time window most suitable for the analysis of brain network activity during aiming, we analyzed correlations between the mean PLV and shooting performance in six frequency bands (Theta to Beta2) and three time windows (Win1 to Win3). As showed in Table 1, correlation coefficients were strongest in the Beta1 band and slightly weaker in the Beta2 band (*r*=−0.45, *P* < 0.01 in Beta1; *r*=−0.38, *P* < 0.05 in Beta2). Correlation coefficients during Win3 were the highest among the three time windows. Consequently, we mainly evaluated the coupling strength and brain network topology in the Beta1 and Beta2 bands during Win 3.

### 3.2. The Relationship between Coupling Strength and Shooting Performance

**Figure 3:**
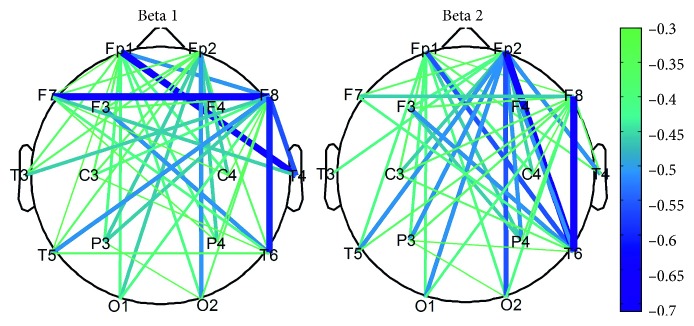
The correlation analysis between functional coupling strength and the shooting performance in Beta1 and Beta2 bands. The color-based lines in the figure represent the correlation coefficient of the significantly correlated connection pair (*P* < 0.05, uncorrected) between each link and shooting performance. The color bar on the right represents the relationship between the correlation coefficient (*r*) and the color. For interpretation of the references to color in this figure legend, the reader is referred to the web version of this article.

Figure 3 shows the correlation analysis between the functional coupling strength in Beta1 and Beta2 bands and shooting performance. The link in the figure represents the functional connection that showed a significant correlation with the shooting performance (*P* < 0.05, uncorrected). The functional coupling strength during the aiming period was negatively correlated with the shooting performance. The lower the functional coupling strength in the Beta1 and Beta2 bands, the better the shooting performance. Furthermore, correlations were the strongest in the right hemisphere (right prefrontal, right frontal, and right temporal regions). Thus, there was an obvious laterality: weaker functional coupling in right brain was associated with better shooting performance.

### 3.3. The Relationship between Network Topology Characteristics and Shooting Performance

Table 2 shows means, standard deviations, and correlation coefficients of the global brain network topological characteristics in Beta1 and Beta2 frequency bands during Win3. As Table 2 shows, the clustering coefficient and the characteristic path length in the Beta1 band significantly correlated with the shooting performance. In the Beta2 band, the clustering coefficient, the characteristic path length, global efficiency, and mean local efficiency significantly correlated with the shooting performance.

**Figure 4:**
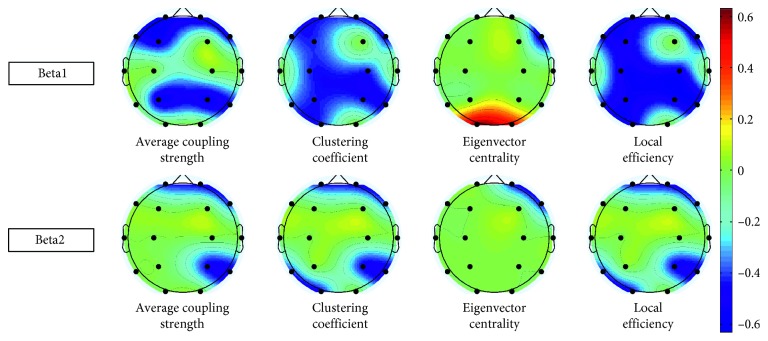
The correlation analyses between local brain network topological characteristics and shooting performance in the Beta1 and Beta2 band. The black nodes indicate electrode position. The color-based regions in the figure represent the correlation coefficient of the significantly correlated connection pair (*P* < 0.05, uncorrected) between each local network topological and shooting performance. Red indicated positive correlations. Blue indicated negative correlations, and color depth represents the correlation coefficient strength. The color bar on the right represents the relationship between the correlation coefficient (*r*) and the color. For interpretation of the references to color in this figure legend, the reader is referred to the web version of this article.

Regarding local brain network topology, we analyzed four characteristics: the average coupling strength, local efficiency, eigenvector centrality, and local clustering coefficients (Figure 4).

In the Beta1 band, average coupling strength, local efficiency, and local clustering coefficients in almost all brain areas significantly negatively correlated with shooting performance. Regarding eigenvector centrality, the O1 and O2 nodes showed significant positive correlations, while the F8 node in the right frontal region showed significant negative correlations with shooting performance.

In the Beta2 frequency band, all four local brain network characteristics negatively correlated with shooting performance. For average coupling strength, local efficiency, and clustering coefficient, only the Fp1, Fp2, F8, P4, T6, O1, and T5 nodes showed significant negative correlations with shooting performance. For eigenvector centrality, only the Fp2 and F8 nodes in the right frontal region showed significant negative correlations with shooting performance.

## 4. Discussion

We collected and analyzed shooting performance data and EEG signals of 40 skilled shooters and found that the shooting performance significantly correlated with the characteristics of EEG brain network during aiming. Particularly, we observed a correlation between the mean PLV and shooting performance only in Beta1 and Beta2 frequency bands. Moreover, functional coupling, global topology, and local topology were also significantly correlated with shooting performance. This may indicate that the neural activity during aiming reflects the shooting state or that the neural activity during aiming affects shooting performance.

### 4.1. Significant Frequency Band and Time Window

Before discussing the correlation between the characteristics and shooting performance, we first determined the most suitable frequency band and time window for analysis.

First, in comparing correlations between the mean PLV and shooting performance in all possibly analyzed frequency bands, we found that a significant correlation between the mean PLV and shooting performance during aiming on Beta1 and Beta2 bands. No significant correlation was discovered in the other frequency bands. In the literature, it has been reported that the Theta band signal appears during sleepiness and is also associated with a negative mood; the Alpha band signal has been reported to be related to whole brain arousal and professional information processing; the Beta band signal has been related to intense emotion and excitement [38, 39]. Therefore, previous papers had mainly studied EEG dynamics within the Alpha band. Research on EEG dynamics in the Beta band is relatively infrequent [15, 17, 19, 40]. In this paper, we examined the correlation between EEG features and shooting performance in all frequency bands using correlation analysis. We found that EEG characteristics in the Beta frequency band were more closely related to shooting performance than in another frequency band.

In previous studies on the EEG functional coupling in shooting, Woo and Kim did not establish a significant correlation between the functional coupling of Beta rhythm and the shooting performance; however, this paper reported a significant correlation between them [41]. There may be set of two reasons for this difference. On the one hand, the experimental conditions of this study differed from those of previous reports. In preceding studies, shooters were mostly asked to adopt a standing position, whereas in the present study, shooters were asked to adopt the prone position. On the other hand, the shooters in this study were not expert shooters, but generally skilled shooters. In contrast, previous papers compared expert to novice shooters. Thus, it is possible that because not all shooters in this study were experts, tension and excitement may have been stronger in our shooters than experts. This may underlie our observation of strong correlations between the EEG characteristics in Beta frequency band and shooting results.

Next, we compared correlation coefficients between EEG dynamics in the Beta frequency band and shooting performance in three time windows. We found that the correlation coefficient in Win3 was larger than in Win1 and Win2. In previous studies, it has been reported as the time of shooting was approaching changes in EEG characteristics became more significant. For example, Kerick et al. found that Alpha2 frequency band power increased most significantly in the left temporal region during the Win3 window. Del Percio et al. found that the ERD/ERS during the Win3 window was the most significant during the aiming [12, 40, 42]. Thus, the results of this study are in line with previous reports. Moreover, these results also point out that not only are the most significant changes in EEG characteristics produced when closest to the shot, but also when the closer the relationship is to the shooting performance. These results show that for the shooters, the closer the time for firing, the more the shooters need to maintain a good mental state.

### 4.2. Laterality and Functional Coupling during Aiming

We analyzed correlations between coupling strength and shooting performance. We found that coupling of Fp1-T6, Fp2-T4, F7-F8, and F8-T6 correlated most significantly with shooting performance. These electrodes were mainly located in the prefrontal, frontal, and right temporal region. In previous studies, it has been reported that the prefrontal and frontal cortices are mainly responsible for advanced task planning and attention; the parietal region is mainly responsible for sensorimotor information processing and the right temporal region is mainly responsible for visual-spatial tasks [14, 43, 44]. Therefore, the strong relationships between these regions and shooting performance indicate that these brain regions and the communication between these regions play a key role in performing the shooting task. Deeny et al. and Del Percio et al. have shown that the level of brain activity of expert shooters during aiming is significantly lower than that of novices, reflecting low energy consumption and high efficiency during aiming [14, 17, 18, 45]. In the present studies, we mainly observed significant negative correlations. In particular, better shooting performance was linked to lower coupling strength. Stronger coupling is involved in higher energy consumption. Therefore, our results are in accordance with the conclusion that the high-level shooters show low consumption and high efficiency phenomenon during aiming.

Compared to functional coupling analyses performed in previous studies, a unique contribution of the analysis performed in the present study is that no regions or electrodes of interest were set a priori. In contrast, Deeny et al. have focused on the connection between the frontal region and other brain regions. Del Percio et al. mainly focused on the connection between the parietal region and other brain regions [17, 19]. In this paper, we analyzed the correlation between shooting performance and all correlation of EEG channels. Therefore, the results of this paper provide a more comprehensive view of the relationship between functional coupling and shooting performance than previous studies. We not only confirm previous conclusions on functional coupling, but also compare these previously reported correlations with other possible functional coupling under the same experimental conditions. We found that among all possible correlations, the correlation between prefrontal, frontal, and right temporal regions are the key areas involved in the shooting.

### 4.3. Brain Network Characteristics during Aiming

Finally, we performed a topological analysis of functional brain networks. For this purpose, we analyzed the correlation coefficients of global and local characteristics of the brain network and shooting performance. In the global network topology analysis, we found that shooters with better shooting performance had lower clustering coefficients, lower local efficiency, shorter characteristic path length, and higher global efficiency during aiming.

Previous brain network topology analyses have shown that the clustering coefficient and mean local efficiency reflect the local information transformation efficiency, while the characteristic path length and global efficiency reflect the global information transformation efficiency [27]. Our results indicate that increased efficiency of local information integration during aiming results in worse shooting performance. In contrast, increased global information integration efficiency facilitates shooting performance.

Interestingly, according to previous studies that investigated the relationship between brain network and cognitive task performance, healthy subjects with higher intelligence quotients and faster response times usually showed higher global information integration efficiency and lower local information integration efficiency, shorter characteristic path length, and lower network clustering coefficients [46–48]. Although these studies investigated cognitive tasks rather than physical activity, according to the results of the paper, it is inferred that the brain network topology, which is significantly related to the performance of cognitive tasks, can also be used to reflect the shooting performance. This phenomenon may indicate that shooting tasks, as a kind of fine motion, are similar to cognitive tasks, and similar neural processes may be shared in the execution of both tasks.

The results of the local network topology analysis provide information about the key brain regions involved in the aiming process. Similar to global network topology, the mean connectivity, local clustering coefficient, and local efficiency of most nodes were negatively correlated with shooting performance. This may indicate that shooters with better shooting performance have lower neural activity in all regions of the brain during aiming. Eigenvector centrality, which reflects the importance of nodes in a network, showed that the right frontal region (F4), the right parietal region (P4), the right frontal region (F8), the left frontal region (F3), and the occipital region (O1 and O2) had significant negative correlations with shooting performance. We speculate that these nodes may be hub nodes and play an important role in the process of aiming. These nodes include the frontal regions, which are regarded as related to advanced information processing; parietal regions, which are related to sensorimotor perception; and the occipital lobes, which related to visual perception and visual-spatial movement [49–53]. The nodes mentioned above have been proposed to be involved in the default network, dorsal attention network, and visual processing network [13, 54]. Taken together, our results indicate that aiming for shooting is a joint and cooperative action of a variety of brain networks that jointly affect shooting performance. However, as a note of caution, it should be considered that our results merely show a significant correlation of eigenvector centrality of these nodes and shooting performance, not that the eigenvector centrality of these nodes is higher than that of other nodes during the aiming period.

Using the PLV network, although we have gained some understanding of the correlation between the EEG brain network activity in Beta band and the shooting performance, there still some limitations persist in this work. First, as the experimental condition of this paper is the rifle shooting of the prone position, it cannot be inferred whether the same result exists for the shooting of other guns (such as pistols) or other positions (such as the standing position). In addition, as for experimental subjects, a significant gap exists in the shooting level between skilled armed police shooters and shooting experts; thus, the conclusion does not necessarily apply to the higher level of shooting athletes. Hence, in the future research, we will investigate the correlation between the shooting performance and EEG network characteristics under other experimental conditions, to obtain more accurate research results.

## 5. Conclusion

In this paper, we collected EEG and shooting performance data from 40 shooters and analyzed correlations between EEG functional network characteristics and shooting performance. Our most significant conclusions are as follows. (1) When comparing the correlation between EEG characteristics and shooting performance in different frequency bands and time windows, the strongest correlations with shooting performance were found in the Beta1 and Beta2 bands during the Win3 time window. This indicates that the closest time window to the firing time has the strongest relation to shooting performance. (2) The correlation analysis between EEG characteristics and shooting performance showed a significant laterality for the functional coupling activity during aiming. The most significant functional coupling related to shooting performance was to be found in the right hemisphere, and the strongest functional coupling was observed between the right prefrontal, frontal, and the right temporal lobe. This indicates that shooting behavior involves the prefrontal cortex, which is responsible for advanced planning, and the right temporal lobe, which is a charge of for the visual-spatial functions. (3) We observed a significant correlation between global and local characteristics of the functional brain network and shooting performance. Our findings suggest that shooters with better shooting performance have higher global brain integration efficiency and lower local information integration efficiency. We also report key nodes that play an important role during the aiming period. Based on this paper, shooting coaches could try to monitor the changes of EEG functional coupling and network topology in the shooting aiming process and could guide shooters in aiming to automatically adjust these EEG characteristics by the use of neural feedback and learn to maintain a good shooting spirit, thereby improving the shooters' performance.

## Figures and Tables

**Table 1:** Mean value (M), standard deviation (SD), and correlation coefficient (*r*) between mean PLV and shooting performance in different frequency bands and different windows. Win1, Win2, Win3, respectively, represents the three time windows during shooting aiming. Win1 represents −6 s to −4 s, Win2 represents −4 s to −2 s, Win3 represents −2 s to 0 s, and 0 s represent the firing time. The above were M ± SD. The down were *r*.

	Theta	Alpha1	Alpha2	Beta1	Beta2
Win1	0.63 ± 0.06	0.65 ± 0.10	0.65 ± 0.11	0.59 ± 0.10	0.58 ± 0.10
	−0.02	−0.06	−0.31	−0.41^*∗*^	−0.28

Win2	0.61 ± 0.06	0.64 ± 0.09	0.65 ± 0.11	0.59 ± 0.10	0.58 ± 0.10
	−0.06	−0.04	−0.30	−0.42^*∗*^	−0.26

Win3	0.60 ± 0.16	0.64 ± 0.10	0.66 ± 0.12	0.60 ± 0.11	0.58 ± 0.11
	−0.05	−0.08	−0.33	−0.45^*∗∗*^	−0.38^*∗*^

^*∗*^ *P* < 0.05, ^*∗∗*^*P* < 0.01.

**Table 2:** The brain network global topological characteristic in Beta1 and Beta2 band. The above is the characteristic's mean value and stander deviation. The down is the characteristic's correlation coefficient between the brain network characteristics and shooting performance. *C* represents the clustering coefficient, *E*_l_ represents the mean local efficiency, *L*_c_ represents the global characteristics characteristic path length, and *E*_g_ represents the global efficiency.

Frequency band		*C*	*E*_l_	*L*_c_	*E*_g_
Beta1	M ± D (*r*)	0.53 ± 0.15 0.45 (^*∗∗*^)	0.49 ± 0.13 −0.45(^*∗∗*^)	0.32 ± 0.19 −0.43(^*∗*^)	3.91 ± 2.31 0.29

Beta2	M ± D (*r*)	0.50 ± 0.14 −0.49 (^*∗∗*^)	0.46 ± 0.13 −0.49 (^*∗∗*^)	0.26 ± 0.18 −0.55 (^*∗∗*^)	4.95 ± 2.77 0.47 (^*∗∗*^)

^*∗*^ *P* < 0.05, ^*∗∗*^*P* < 0.01.

## Data Availability

The data “PLV_ Matrix.mat” is the subjects' functional coupling matrix-based PLV; the data format is 16 × 16 × 2 × 40, which represents the channel × channel × frequency band × subject; frequency bands include Beta1 and Beta2 band. The data “Shooting Performance.mat” is each subject's average shooting performance of 55 shots score.

## Additional Points

EEG characteristics in Beta1 and Beta2 bands during WIN3 showed the strongest correlations with shooting performance among all frequency bands and time windows. Functional coupling between the prefrontal, frontal, and right temporal brain regions during the aiming period was the most negatively correlated with the shooting performance. Shooters with better shooting performance showed higher global brain integration efficiency and lower local information efficiency.
